# Effects of MHD and porosity on entropy generation in two incompressible Newtonian fluids over a thin needle in a parallel free stream

**DOI:** 10.1038/s41598-020-76125-y

**Published:** 2020-12-18

**Authors:** Farhad Ali, Anees Imtiaz, Waqar A. Khan, Ilyas Khan, Irfan A. Badruddin

**Affiliations:** 1 Computational Analysis Research Group, Ton Duc Thang University, Ho Chi Minh City, Vietnam; 2 Faculty of Mathematics and Statistics, Ton Duc Thang University, Ho Chi Minh City, Vietnam; 3 Department of Mathematics, City University of Science and Information Technology, Peshawar, Khyber Pakhtunkhwa Pakistan; 4 Department of Mechanical Engineering, College of Engineering, Prince Mohammad Bin Fahd University I, P.O. Box 1664, Al Khobar, 31952 Kingdom of Saudi Arabia; 5 Department of Mathematics, College of Science Al-Zulfi, Majmaah University, Al-Majmaah, 11952 Saudi Arabia; 6 Department of Mechanical Engineering, College of Engineering, King Khalid University, Abha, 61421 Saudi Arabia

**Keywords:** Applied mathematics, Computational science

## Abstract

This article is devoted to studying Magnetohydrodynamic (MHD)'s combined effect and porosity on the entropy generation in two incompressible Newtonian fluids over a thin needle moving in a parallel stream. Two Newtonian fluids (air and water) are taken into consideration in this study. The viscous dissipation term is involved in the energy equation. The assumption is that the free stream velocity is in the direction of the positive *x-axis*—(axial direction). The thin needle moves in the same or opposite direction of free stream velocity. The reduced similar governing equations are solved numerically with the help of shooting and the fourth-order Runge–Kutta method. The expressions for dimensionless volumetric entropy generation rate and Bejan number are obtained through using similarity transformations. The effects of the magnetic parameter, porosity parameter, Eckert number, Bejan number, irreversibility parameter, Nusselt number, and skin friction are discussed graphically in detail for and taken as Newtonian fluids. The results are compared with published work and are found in excellent agreement.

## Introduction

The second law of thermodynamics is more consistent and efficient than the first law due to the constraints of the first law's proficiency in thermal systems. The first law is also known as the law of conservation of energy and discusses the quantity of energy but not quality. In practical applications such as in engineering and other related science, the quality and quantity both matter, so for this reason, the second law is more general and reliable. The second law of thermodynamics is employed to reduce the irreversibilities^1,2^. The process of degrading the available systems work is known as Entropy generation^3^. The source of entropy generation is heat and mass transfer, viscous dissipation, and many more because the entropy generation is applied to improve the system^4^. Primarily, the entropy generation with the second law has been investigated by Bejan^5^ and originate that the temperature and velocity gradient are responsible for creating entropy generation in fluid flow. Afterward, this topic attracted many researchers, and they have used this idea in different world problems due to its vast applications^6–10^.

In the latest times, the magnetic field observation with heat transfer has an essential role in sciences, mainly in physics, engineering, and remedies, such as MHD turbines, sand boundary layer control, and pumps, etc. Physically, the MHD plays an essential position in regulating the momentum and heat switch in one kind of fluid. It is well worth citing that the MHD actively modified the heat transfer's overall performance within the flow to rearrange their consideration within the fluid. The concept of MHD was introduced by Hannes^11^, for which he got a Nobel prize, and then many researchers used the idea of MHD in various problems and obtained tremendous results in Medical sciences and engineering^12,13^. The concept of a thin needle with MHD and entropy generation is also an important topic and needs to be investigated. The thin needle formed like a paraboloid body whose axis in the path of the incident flow. Its diameter is of the same order as of velocity, or thermal boundary layers developed. Hence the axisymmetric type of structure allows a similarity solution to withdrawal and qualify to study the problem in detail. Axisymmetric flow and heat transport over a thin transferring needle has been investigated by many researchers in the presence of various flow conditions. Wang et al.^14^ numerically and theoretically investigated the pin-2 BEC in an optical lattice. The exact soliton solutions and nonlinear modulation instability in spinor Bose–Einstein condensates are obtained by Li et al*.*^15^. Ji et al*.*^16^ investigated the dynamic creation of fractionalized half-quantum vortices in Bose–Einstein condensates of sodium atoms.

The shape of a needle is much like a paraboloid of revolution parallel to go with the flow. Initially, the boundary layer flow by a thin needle has been examined by Lee^17^. Ishak et al*.*^18^ discussed the thin needle in a parallel free stream boundary layer flow and obtained the dual solutions when the needle and free stream move in the opposite direction. The boundary layer flow with nanofluid over a thin needle has been investigated by Soid et al*.*^19^. Hayat et al*.*^20^ investigated the thin needle, introducing carbon nanofluid with variable heat flux, and used the shooting method for the solution of the problem. The mixed convection flows with heat transfer in a moving thin needle with nanofluid for assisting and opposite cases investigated by Salleh et al*.*^21^. Ahmad et al*.*^22^ examined the mixed convection boundary layer flow along a thin vertical needle with nanofluid and obtained the solution with the help of the Finite difference scheme and Keller box method. The classical model for forced convection flow with heat transfer and wall temperature using Copper and Aluminum Oxide Nanoparticles and -based fluid is analyzed by Grosan et al*.*^23^. Ahmad et al.^24^ discussed Buongiorno’s Model in axially moving the thin needle with nanofluid and two slip velocity mechanism Brownian motion and thermophoresis. The entropy generation with heat transfer in the presence of Rosseland radiation for a boundary layer flow over a thin needle has been examined by Afridi et al*.*^25^. Khan et al*.*^26^ recently discussed the entropy generation for two non-Newtonian fluids with first and second law analysis for a moving thin needle and obtained the solution by similarity transformation. From all, the above-discussed problems on thin needle focused on the heat transfer analysis and some recent on entropy generation. Still, no devotion has been awarded to the study of boundary layer flow with MHD and porosity over a thin needle moving in a parallel stream. Even not a single paper is published on entropy generation for thin needle problem with porosity and MHD. Therefore, this study aims to perform such an analysis. Suitable transformation is applied to convert basic governing equations to self-similarity equations. The shooting technique, along with the Runge–Kutta technique, is used in obtaining numerical solutions. The calculated velocity and temperature gradients are used to calculate the entropy generation rate. The impact of various physical parameters on velocity, temperature distribution, entropy production, and Bejan number is shown through graphs and discussed.

### Mathematical formulation

**Figure 1:**
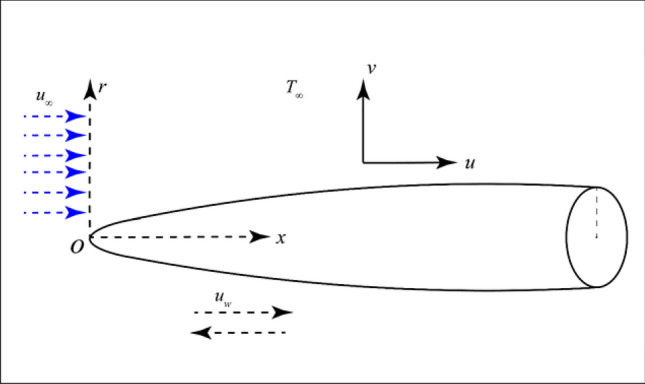
Physical configuration of the problem.

A steady flow of two incompressible non-Newtonian fluids has been considered over a thin needle moving with velocity $u_{w}$ in a parallel free stream. The transverse magnetic field and porosity have also been taken into account. The needle thickness is comparatively less than the momentum and thermal boundary layer. The radius of the needle is described by $r = R\left( x \right)$ where *r* and *x* represent the radial and Cartesian coordinates. The physical configuration of the problem is given in Fig. 1

Under the above assumptions, the boundary layer equations in cylindrical coordinates are given by^18,19,26–29^:

1 $$ \frac{\partial }{\partial x}\left( {ru} \right) + \frac{\partial }{\partial r}\left( {rv} \right) = 0, $$

2 $$ u\frac{\partial u}{{\partial x}} + v\frac{\partial u}{{\partial r}} = \frac{v}{r}\frac{\partial }{\partial r}\left( {r\frac{\partial u}{{\partial r}}} \right) - \left( {\frac{{\sigma B_{0}^{2} }}{\rho } + \frac{\nu \phi }{k}} \right)u, $$

3 $$ u\frac{\partial T}{{\partial x}} + v\frac{\partial T}{{\partial r}} = \frac{\alpha }{r}\frac{\partial }{\partial r}\left( {\frac{\partial T}{{\partial r}}} \right) + \frac{\nu }{{\left( {c_{p} } \right)}}\left( {\frac{\partial u}{{\partial r}}} \right)^{2} , $$

4 $$ \dot{S}^{\prime\prime\prime}_{gen} = \frac{\kappa }{{T_{\infty }^{2} }}\left( {\left( {\frac{\partial T}{{\partial r}}} \right)^{2} + \left( {\frac{\partial T}{{\partial x}}} \right)^{2} } \right) + \frac{\mu }{{T_{\infty } }}\left( {2\left( {\left( {\frac{\partial v}{{\partial r}}} \right)^{2} + \left( {\frac{\partial u}{{\partial x}}} \right)^{2} } \right)} \right) + \left( {\left( {\frac{\partial u}{{\partial r}}} \right) + \left( {\frac{\partial v}{{\partial x}}} \right)} \right)^{2} . $$

The corresponding initial and boundary conditions are:

5 $$ \left. \begin{gathered} u = u_{w} ,\,\;v = 0,\,\,T = T_{0,} {\text{at }}r = R\left( x \right) \hfill \\ u \to u_{\infty } ,\,\,\,T \to T_{\infty ,} {\text{ when }}r \to \infty \hfill \\ \end{gathered} \right\} $$

Equation (1) represents the continuity equation in cylindrical coordinates. The momentum equation for the flow behavior of the thin needle is given in Eq. (2). The temperature distribution in cylindrical as well as cartesian coordinates is described in Eq. (3), while Eq. (4) represents the entropy generation.

### The solution of the model

For the solution of the momentum equation of the thin needle, the similarity variables will be used by^18,30^:

6 $$ \,\psi (x,\,r) = \nu x\,f(\eta ),\eta = \frac{{Ur^{2} }}{\nu x},\,u = \frac{1}{r}\frac{\partial \psi }{{\partial r}}{, }v = - \frac{1}{r}\frac{\partial \psi }{{\partial x}},\,U = u_{w} + u_{\infty } $$

Using Eq. (6), into the Eq. (1), the continuity equation is identically satisfied and the governing equations of velocity with the transformed boundary conditions yield:

7 $$ \eta f^{\prime\prime\prime} + f^{\prime\prime} + \frac{1}{2}ff^{\prime\prime} - \frac{1}{4}\left( {M + \frac{1}{K}} \right)f^{\prime} = 0 $$

8 $$ \left. \begin{gathered} f\left( \eta \right) = \frac{a\varepsilon }{2},\,f^{^{\prime}} \left( \eta \right) = \frac{\varepsilon }{2},\theta \left( \eta \right) = 1\,\,{\text{when }}\eta = a \hfill \\ f^{^{\prime}} \left( \eta \right) = \frac{1 - \varepsilon }{2},\theta \left( \eta \right) = 0\,{\text{when }}\eta \to \infty \hfill \\ \end{gathered} \right\} $$

Such that the primes denote the derivatives of $f$ w.r.t $\eta$. $r = R\left( x \right) = \sqrt {\frac{\nu xa}{U}} ,\varepsilon = \frac{{u_{w} }}{U}$ shows the dimensionless velocity and $K = \frac{k}{\nu \phi }$ is the porosity parameter.

### Skin friction

The expression for the dimensional skin friction is given by^18^

9 $$ C_{f} = \left. {\frac{\mu }{{\rho \left( U \right)^{2} }}\left( {\frac{\partial u}{{\partial r}}} \right)} \right|_{r = R\left( x \right)} $$

Using Eq. (6), the non-dimensional form of Eq. (9) is written as

10 $$   \left( {{\text{Re}}_{x} } \right)^{1/2} C_{f} = 4\sqrt a f \left( a \right) $$

where ${\text{Re}}_{x} = \frac{Ux}{\nu }$ shows the local Reynolds number.

### Temperature distribution

For the solution of the energy equation as described in Eq. (3), the similarity solution^1^ will be used as

11 $$ \,\theta \left( \eta \right) = \frac{{T - T_{\infty } }}{{T_{w} - T_{\infty } }} $$

From Eq. (11) the transformed form of Eq. (3) along with the boundary conditions are given by;

12 $$   \eta \theta^{\prime\prime} + \left( {1 + 0.5\Pr f} \right)\theta ^{\prime} + 4\Pr \eta {\text{Ec}}\left( {f} \right)^{2} = 0, $$

13 $$ \left. \begin{gathered} \theta \left( \eta \right) = 1\,\,{\text{when }}\eta = a \hfill \\ \theta \left( \eta \right) = 0\,{\text{when }}\eta \to \infty \hfill \\ \end{gathered} \right\} $$

where $$\begin{gathered} \Pr = \frac{\nu }{\alpha },Ec = \frac{{U^{2} }}{{c_{p} \left( {T_{w} - T_{\infty } } \right)}}, \hfill \\ \,\,\,\,\,\,\,\,\,\,\, \hfill \\ \end{gathered}$$ shows the Prandtl and Eckert numbers, respectively.

#### Nusselt number

The expression for the dimensional Nusselt number is given by^19^

14 $$ {\text{Nu}} = \frac{{xq_{w} }}{{\kappa \left( {T_{w} - T_{\infty } } \right)}};\quad q_{w} = - \kappa \left. {\frac{\partial T}{{\partial r}}} \right|_{r = R\left( x \right)} $$

Using Eq. (11), the non-dimensional form of Eq. (14) is written as

15 $$ \left( {{\text{Re}}_{x} } \right)^{ - 1/2} {\text{Nu}}_{x} { = } - 2\sqrt a \,\theta^{/} \left( a \right), $$

### Entropy generation

The expression for the entropy generation for an incompressible non-Newtonian fluid in cylindrical coordinates using the boundary layer approximations is given by

16 $$ \dot{S}^{\prime\prime\prime}_{gen} = \frac{\kappa }{{T_{\infty }^{2} }}\left( {\left( {\frac{\partial T}{{\partial r}}} \right)^{2} } \right) + \frac{\mu }{{T_{\infty } }}\left( {\left( {\frac{\partial u}{{\partial r}}} \right)^{2} } \right) + \frac{{\sigma B_{0}^{2} }}{{T_{\infty } }}u^{2} + \frac{\mu }{{kT_{\infty } }}u^{2} , $$

where the first term shows the entropy generation rate due to heat transfer $\dot{S}^{\prime\prime\prime}_{h}$, the second term shows the entropy generation rate due to fluid friction ${{\dot{S}^{\prime\prime\prime}}}_{{\text{f}}}$, the third term shows the entropy generation rate due to the magnetic field ${{\dot{S}^{\prime\prime\prime}}}_{{\text{m}}}$, and the last term shows the entropy generation rate due to porous medium ${{\dot{S}^{\prime\prime\prime}}}_{{{\text{pm}}}}$.

17 $$ {{\dot{S}^{\prime\prime\prime}}}_{{{\text{gen}}}} {{ = \dot{S}^{\prime\prime\prime}}}_{{\text{h}}} + {{\dot{S}^{\prime\prime\prime}}}_{{\text{f}}} + {{\dot{S}^{\prime\prime\prime}}}_{{\text{m}}} + {{\dot{S}^{\prime\prime\prime}}}_{{{\text{pm}}}} $$

In dimensionless form, the total entropy generation rate can be written as

18 $$   Ns = \frac{{\dot{S}^{\prime\prime\prime}_{gen} \,}}{{\left( {4k_{f} /x^{2} } \right)\Omega_{T} }} = \eta {\text{Re}}_{x} \Omega_{T} \left( {\theta ^{\prime}} \right)^{2} + 4\eta {\text{Re}}_{x} Br\left( {f} \right)^{2} + Br{\text{Re}}_{x} \left( {M + \frac{1}{K}} \right)\left( {f^{\prime}} \right)^{2} $$

where $Br = \frac{{\mu U^{2} }}{{k_{f} \left( {T_{w} - T_{\infty } } \right)}},\frac{1}{K} = \frac{x\nu }{{Uk}}$ represents the Brinkman number, modified Brinkman number, and modified porosity.

Concerning the entropy generation analysis of convective heat transfer problems, Bejan^1,2^ represented the expression of irreversibility distribution ratio as follows

19 $$ \Phi = \frac{{\dot{s}^{\prime\prime\prime}_{prod,frc} }}{{\dot{s}^{\prime\prime\prime}_{prod,\Delta T} }} $$

It is noteworthy to mention that when Φ > 1, the fluid friction irreversibility $Ns_{f}$ plays a major role. Otherwise, the heat transfer irreversibility $Ns_{h}$ is dominant. When Φ = 1, the improvement secondary to heat transfer ($Ns_{h}$) and to fluid friction ($Ns_{f}$) are equal.

Equation (19) will become

20 $$   \Phi = \frac{{4Br_{1} \left( {f} \right)^{2} + \frac{Br}{{\Omega_{T} \eta }}\left( {M + \frac{1}{{k_{1} }}} \right)\left( {f^{\prime}} \right)^{2} }}{{\left( {\theta^{/} } \right)^{2} }} $$

The irreversibility ratio also known as Bejan number is

21 $$ Be = \frac{{\dot{S}^{\prime\prime\prime}_{prod,\Delta T} }}{{\dot{S}^{\prime\prime\prime}_{prod} }} = \frac{{\frac{\kappa }{{T_{\infty }^{2} }}\left( {\frac{\partial T}{{\partial r}}} \right)^{2} }}{{\frac{\kappa }{{T_{\infty }^{2} }}\left( {\frac{\partial T}{{\partial r}}} \right)^{2} + \frac{\mu }{{T_{\infty } }}\left( {\frac{\partial u}{{\partial r}}} \right)^{2} }} $$

From the similarity transformation, Eq. (21) becomes

22 $$   Be = \frac{{\theta^{/2} }}{{\theta^{/2} + 4Br_{1} \left( {f} \right)^{2} + \frac{Br}{{\Omega_{T} \eta }}\left( {M + \frac{1}{{k_{1} }}} \right)\left( {f^{\prime}} \right)^{2} }} $$

Be = 1 is the limit at which the irreversibility is due to heat transfer only, and *Be* = 0 is the limit at which the irreversibility is due to fluid friction only. Irreversibility due to heat transfer dominates when *Be* >  > $\frac{1}{2}$, while Be <  < $\frac{1}{2}$ shows that irreversibility due to fluid friction dominates.

### Limiting solution

By considering H $\to 0$ in Eq. (7), the solution is reduced to the results obtained by Soid et al.^19^ by ignoring the nanofluid terms and is given by

23 $$ 2\left( {f^{\prime\prime\prime} - \eta f^{\prime\prime}} \right) + ff^{\prime\prime} = 0 $$

The expressions for the Nusselt number and skin friction, obtained in Eqs. (10) and (15), are identical to the expression obtained by Soid et al.^19^ by absenting the nanofluid terms. The values of skin friction are compared in Table 1, with the available data for a particular case.

**Table 1:** Comparison of skin friction along a thin needle with the existing data for different values of *a*.

*a*	Ishak et al*.*^18^ λ = 0	λ = − 1	Soid et al*.*^19^ λ = 0	λ = − 1	Present results λ = 0	λ = − 1
0.01	8.492	26.602	8.491	26.600	8.531	26.628
0.1	1.289	3.716	1.289	3.704	1.315	3.763
0.2	–	–	0.752	2.005	0.774	2.075

## Graphical results and discussion

In this problem, the entropy generation with MHD and porosity for two non-Newtonian fluids have been discussed for a moving thin needle. The comparison of the skin friction along a thin needle for different needle sizes has been reported in Table 1. As the needle's size increases, the area of the needle increases, which increases the skin friction. The present results are found in good agreement with the published work.

**Figure 2:**
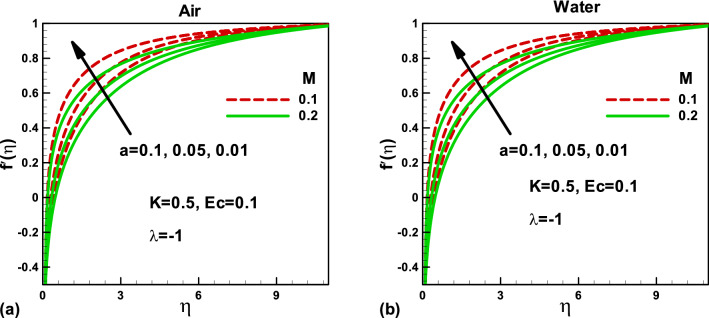
Effects of the magnetic parameter on dimensionless velocity for **(a)** and **(b)** when the free stream is moving in the positive x-axis and the needle is moving in the opposite direction.

**Figure 3:**
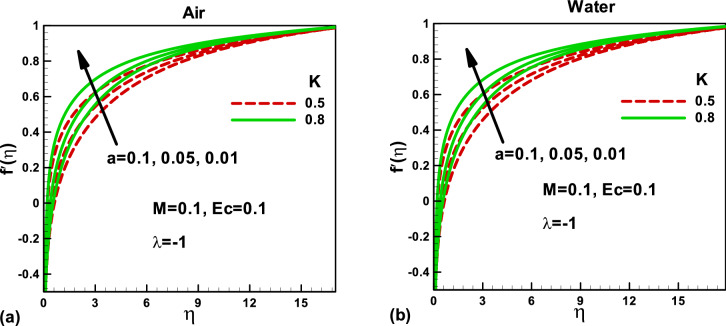
Effects of porosity parameter on dimensionless velocity for **(a)** and **(b)** when the free stream is moving in the positive x-axis and the needle is moving in the opposite direction.

**Figure 4:**
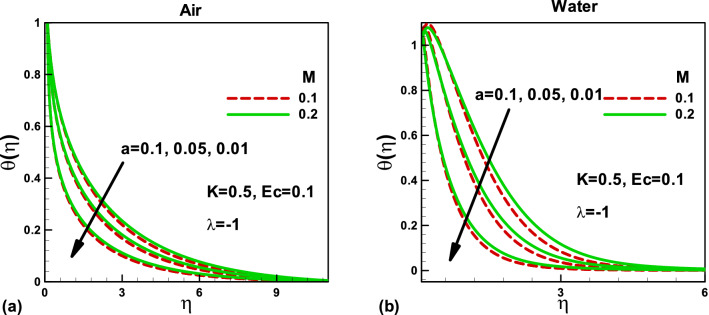
Effects of the magnetic parameter on dimensionless temperature for **(a)** and **(b)** when the free stream is moving in the positive x-axis and the needle is moving in the opposite direction.

**Figure 5:**
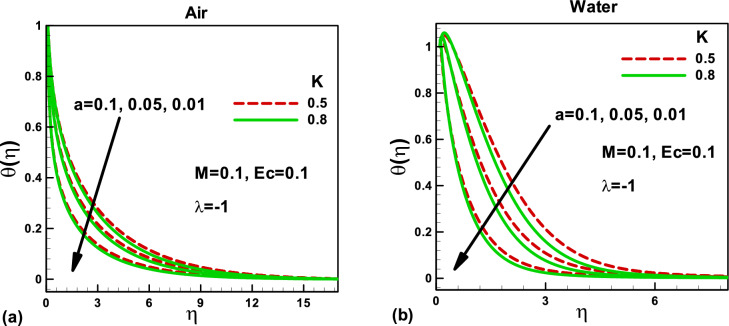
Effects of porosity parameter on dimensionless temperature for **(a)** air and **(b)** water, when the free stream is moving in the positive x-axis and the needle is moving in the opposite direction.

**Figure 6:**
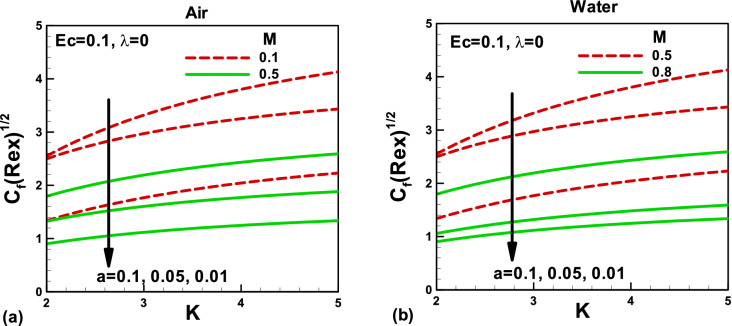
Effects of magnetic and porosity parameters on skin friction for **(a)** air and **(b)** water, when the needle is fixed in a moving fluid.

**Figure 7:**
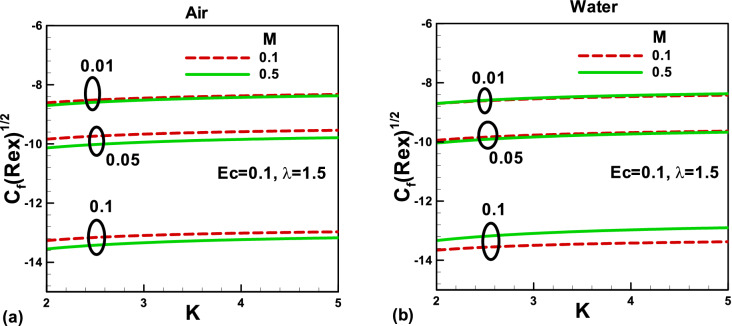
Effects of magnetic and porosity parameters on skin friction for **(a)** air and **(b)** water, when the free stream is moving along the negative x-axis and the needle is moving in the opposite direction.

**Figure 8:**
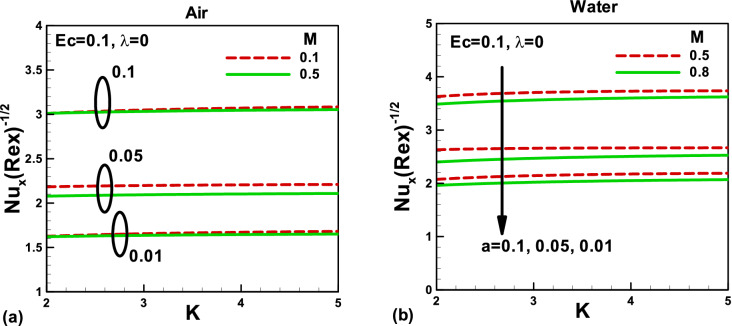
Effects of magnetic and porosity parameters on Nusselt number for **(a)** air and **(b)** water, when the needle is fixed in a moving fluid.

**Figure 9:**
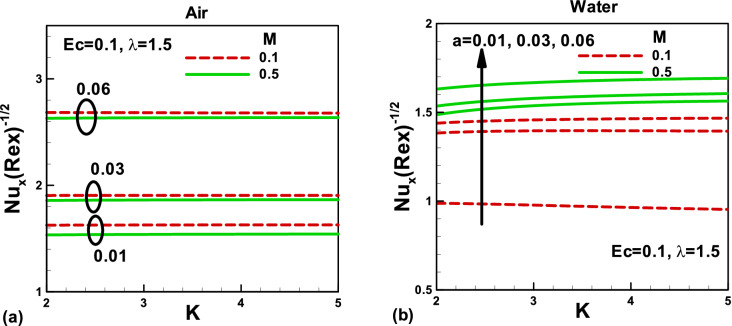
Effects of magnetic and porosity parameters on Nusselt number for **(a)** air and **(b)** water, when the free stream is moving along the negative x-axis and the needle is moving in the opposite direction.

**Figure 10:**
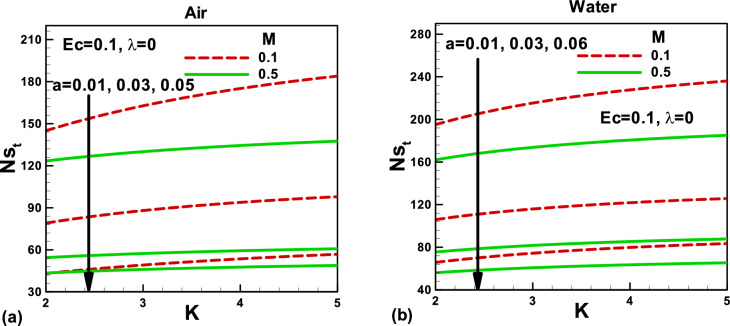
Effects of magnetic and porosity parameters on total entropy generation rate for **(a)** air and **(b)** water, when the needle is fixed in a moving fluid.

**Figure 11:**
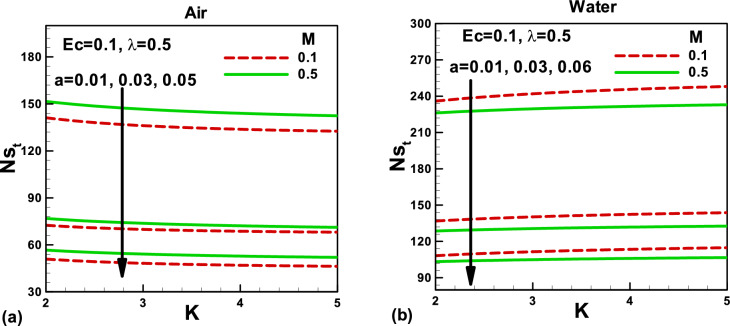
Effects of magnetic and porosity parameters on total entropy generation rate for **(a)** air and **(b)** water, when both needle and fluid are moving in the same direction.

**Figure 12:**
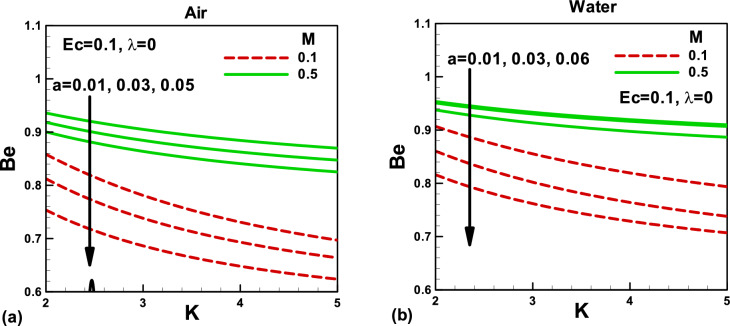
Effects of magnetic and porosity parameters on Bejan number for **(a)** air and **(b)** water, when the needle is fixed in a moving fluid.

**Figure 13:**
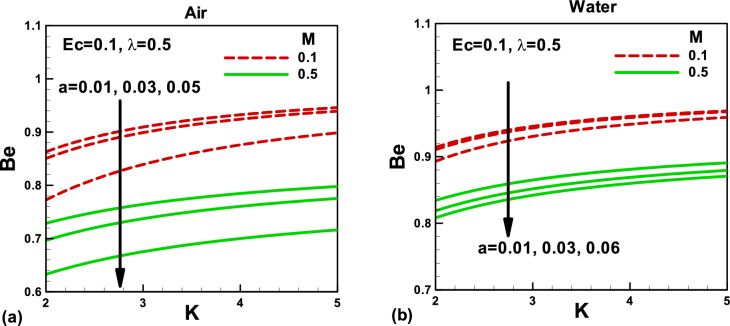
Effects of magnetic and porosity parameters on Bejan number for **(a)** air and **(b)** water, when both needle and fluid are moving in the same direction.

**Figure 14:**
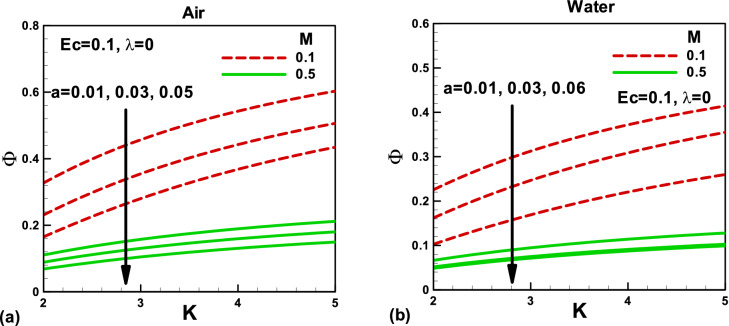
Effects of magnetic and porosity parameters on irreversibility distribution ratio for **(a)** air and **(b)** water, when the needle is fixed in a moving fluid.

**Figure 15:**
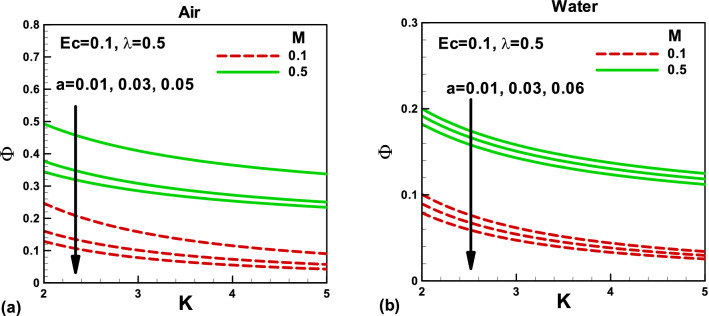
Effects of magnetic and porosity parameters on irreversibility distribution ratio for **(a)** air and **(b)** water, when both needle and fluid are moving in the same direction.

**Figure 16:**
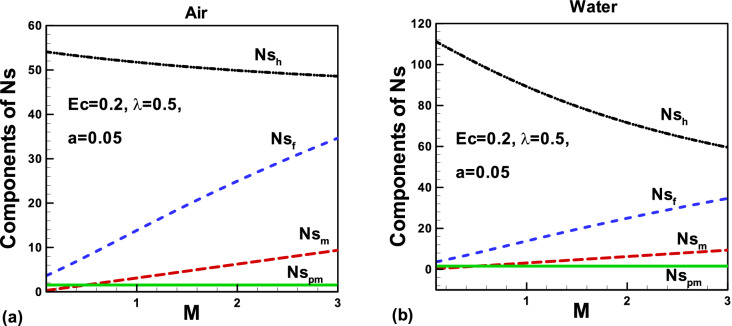
Effects of the magnetic parameter on components of entropy generation rate for **(a)** air and **(b)** water, when both needle and fluid are moving in the same direction.

The physical configuration of the problem is given in Fig. 1. For the physical behavior and interpretations, the Figs. 2, 3, 4, 5, 6, 7, 8, 9, 10, 11, 12, 13, 14, 15 and 16 have been drawn for two Newtonian fluids, air, and water, to show the effect of various physical parameters on the dimensionless velocity, temperature, entropy generation rate, and Bejan number. The effects of magnetic parameter *M* and porosity *K* have been shown for dimensionless velocity in Figs. 2 and 3 when the needle is moving opposite to the free stream's positive x-axis. Figure 2a,b present the magnetic field's effect on the dimensionless velocity for different needle sizes. Due to the magnetic field, a resisting force, Lorentz force, is acted on the needle, which reduces the dimensionless velocity of each fluid. Consequently, the velocity boundary layer thickness decreases, which reduces the shear stress. The dimensionless velocity, inside the boundary layer increases with decreasing needle size in both cases, as shown in Fig. 2a,b. Figure 3a,b display the porosity parameter's effects on the dimensionless velocity for different needle sizes. The porous medium opposes the flow. An increase in the porosity parameter increases the velocity boundary layer thickness, which decreases the resistance to the fluid flow. The needle size also helps in reducing the dimensionless velocity in both cases.

For the dimensionless temperature, the effects of magnetic parameter *M* and porosity parameter *K* are depicted in Figs. 4 and 5. From Fig. 4a,b, it has been observed that augmenting *M*, the fluid temperature increases due to reducing the thickness of the boundary layer flow according to the Lorentz theory. Physically, the magnetic field reduces the velocity in the boundary layer, which increases the temperature. An increase in the surface area due to increased needle size increases the dimensionless temperature in both cases. However, in Figs. 5a,b, the fluid temperature decreases by increasing the porosity due to the increase in the boundary layer thickness. These two effects have been validated, as discussed by Shah et al*.*^31^. In both cases, a decrease in the needle size helps in reducing the temperature of both fluids.

The skin friction variation with the porosity parameter is depicted in Fig. 6a for air and in Fig. 6b for water when the needle is fixed in a moving fluid. As explained before, the porous medium resists the flow and decreases the fluid velocity in both cases. As a result, the skin friction increases in both cases. The impacts of the magnetic field and needle size on the skin friction are presented in Fig. 6a,b. An increase in the needle size increases the surface area, and as a result, the skin friction increases in both cases. The magnitude of the skin friction decreases with an increase in *M.* Physically; this is due to an increase in the Lorentz force, which decreases the velocity boundary layer thickness and the hydraulic resistance to flow. Consequently, the skin friction decreases with an increase in the magnetic field. Figure 7a,b also show the variation of skin friction with the porosity and magnetic parameters for different needle sizes, but in this case, the needle is not static; it is moving in the opposite direction. It reveals that by decreasing the needle's size, the skin friction increases for both fluids due to a rise in the momentum boundary layer thickness and fall in the shear stresses. For smaller needle size, the magnetic field's effect on the skin friction is negligible and increases with an increase in the magnetic field in both cases. The effects of needle sizes are found to be the same as in Fig. 6.

When the needle is fixed in a moving fluid, the variation of Nusselt number with magnetic and porosity parameters for different needle sizes is presented in Fig. 8a,b. It is important to note that the Nusselt number decreases with a decrease in the needle size. Physically, this is due to a reduction in the surface area with the needle size in both cases. For larger needle size, no noticeable effect of the magnetic field on the Nusselt number could be found in the case of air. However, this effect becomes visible in the case of water. Also, the porosity parameter makes no visible effect on the Nusselt number. On the other side, when the free stream moves along the negative x-axis and the needle moves in the opposite direction, the effects of needle size are elaborated in Fig. 9a,b for air and water, respectively. Due to increased surface area with needle size, the Nusselt number increases in both cases, for smaller values of the magnetic parameter, no appreciable effect on Nusselt number for both fluids. However, for water, the Nusselt number increases with both porosity and magnetic field.

Figures 10 and 11 depict the impacts of pertinent parameters on the total entropy generation rate for air and water when the needle is fixed in a moving fluid. According to the second law of thermodynamics, entropy generates due to several irreversibilities. The total entropy generation rate comprises these irreversibilities, as shown in Eq. (16). The variation of the total entropy generation rate with the magnetic and porosity parameters is displayed in Fig. 10a,b for the selected needle sizes. An increase in the needle size increases the surface area of a thin needle, which reduces velocity and temperature gradients, see Figs. 2, 3, 4 and 5. Accordingly, the total entropy generation rate reduces with increasing needle size. Similarly, the magnetic parameter facilitates reducing the total entropy generation rate. However, the porosity parameter tends to resist the fluid flow and increases the total entropy generation rate. The effects of the same parameters on the total entropy generation rate are described in Fig. 11. In this case, the porosity parameter demonstrates almost negligible effects in both cases. However, water indicates higher entropy generation rates. The Bejan number signifies the importance of thermal irreversibility in the total irreversibility. The thermal irreversibility leads when $Be > 0.5$ the irreversibility due to viscous dissipation, porous medium, and the magnetic field is influential when $Be < 0.5$. In Fig. 12a,b, the Bejan number is found to be greater than 0.5, which shows that the thermal irreversibility plays a major role in both cases. It is important to note that when the needle is fixed in a moving fluid, the Bejan number increases with an increasing magnetic field in both cases. However, Bejan number decreases needle size due to an increase in surface area. On the other side, when both needle and fluid move in the same direction, Bejan number decreases with an increasing magnetic field in both cases, as shown in Fig. 13a,b for both fluids.

The irreversibility distribution ratio φ compares the viscous dissipation irreversibility with the thermal irreversibility. It is important to note that, when the needle is fixed in a moving fluid, the needle size reduces the irreversibility distribution ratio, Fig. 14a,b. Whereas, the magnetic field and porosity parameter increases the viscous irreversibility, which increases the irreversibility ratio. On the other side, when both needle and fluid are moving in the same direction, both needle size and porosity parameter reduce the irreversibility distribution ratio due to an increase in the thermal irreversibility, Fig. 15a,b. However, the magnetic field increases the irreversibility ratio due to an increase in the viscous irreversibility in both cases. The comparison shows that the irreversibility distribution ratios are lower for water than air.

The variation of several components of the total entropy generation rate with the magnetic field is displayed in Fig. 16a,b. It can be seen that the thermal irreversibility is higher than any other irreversibility due to an increase in temperature with an increasing magnetic field. As expected, other irreversibility increases at different rates in both cases. The comparison shows that water offers higher thermal reversibility than air.

## Conclusion

This work determines MHD and Porosity's effects on Entropy Generation over thin needle in a Parallel Free Stream for Two Incompressible Newtonian fluids. The following are the concluding remarks of the present study:

1. Skin friction enhances by increasing the size and area of the needle.
2. The magnetic parameter retards the velocity of both the fluids.
3. Increasing the porosity parameter enhances the velocity boundary layer thickness, decreasing the resistance to the fluid flow.
4. The fluids temperature decreases by decreasing the needle size and enhancing the *M* and *K*
5. For a fixed needle, the heat transfer rate decreases, while for increasing M and K's value*,* the heat transfer rate increases for the moving needle opposite direction to the free stream.
6. The total entropy generation rate in water is more than the air.
